# Anti-HPA-1b Mediated Posttransfusion Purpura: A Case Report

**DOI:** 10.1155/2013/568364

**Published:** 2014-01-16

**Authors:** O. P. Arewa, S. Nahirniak, G. Clarke

**Affiliations:** ^1^Department of Laboratory Medicine & Blood Bank, Arar Central Hospital, Arar, Saudi Arabia; ^2^Department of Laboratory Medicine & Pathology, University of Alberta Hospital, Edmonton, AB, Canada

## Abstract

Posttransfusion purpura (PTP) is an uncommon, but potentially fatal, transfusion reaction characterized by profound thrombocytopenia and bleeding. PTP is caused by alloimmunization to human platelet specific antigens following blood component transfusion. Although there is evidence of a wide serological spectrum of culprit antibodies implicated, Anti-human-platelet-antigen- (HPA-) 1a is the most common antibody in cases reported. We report a case of posttransfusion purpura in an African American. The patient was negative for HPA-1a antibodies, but anti-HPA-1b was identified with a platelet phenotype of HPA-1a/HPA-1a. Although less common, HPA-1b antibody may be an important consideration in posttransfusion purpura diagnosed in patients of African descent.

## 1. Introduction

Posttransfusion purpura (PTP) is an uncommon, but potentially life threatening, transfusion reaction characterized by profound thrombocytopenia and bleeding caused by alloimmunization to human platelet antigens following transfusion of a cellular blood component. The incidence estimates in the literature are quite variable, between 1 : 50,000 and 100,000 transfusions, and the majority of cases occur in multiparous women [1]. The youngest patient reported to have developed PTP is a 16 yr old female of African descent with no history of pregnancy [2]. The diagnosis of PTP is difficult. There is little awareness of this disorder which may result in underrecognition; the patients being transfused are often critically ill, and alternative explanations for thrombocytopenia such as infection, drugs, and DIC are part of the differential diagnosis. With an onset typically between 4 and 12 days from the index transfusion, prompt recognition and an early institution of management are crucial to improving patient morbidity and mortality. Although there is evidence of a wide serological spectrum of culprit antibodies implicated in the pathogenesis of the condition, antibodies directed against human-platelet-antigen-1a (HPA-1a) is the most common specificity reported globally. The literature is generally sparse on cases of PTP against its antithetical antigen HPA-1b as the primary specificity. A single nucleotide polymorphism on chromosome 17q21.32 results in substitution of the amino acid proline for leucine at position 33 of GPIIIa to determine HPA-1a and HPA-1b status [3]. The following case report illustrates a case of posttransfusion purpura secondary to anti-HPA-1b in an African American patient managed in our facility.

## 2. Case Presentation

**Figure 1:**
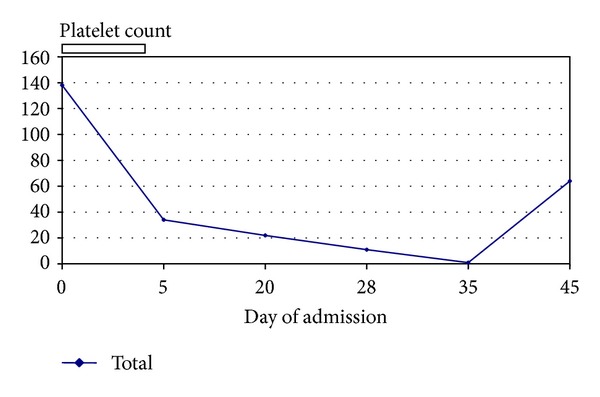
Chart showing trend of platelet count.

A 24-year-old man of African descent was originally admitted to an outlying hospital in cardiogenic shock, secondary to viral myocarditis. He rapidly deteriorated and was admitted into the intensive care unit following development of acute renal failure. He was subsequently dialysed. His renal function gradually improved on dialysis while his cardiac function also significantly improved with the ejection fraction increasing from 20% on admission to 40%. About five days into admission, his hemoglobin was noted to have decreased gradually from the initial value of 148 g/L to 84 g/L despite packed red cell transfusions (Table 1). The platelet count similarly decreased progressively despite multiple platelet transfusions. Blood cultures were negative. The virology workup for cytomegalovirus, EBV, parvovirus B19, and HIV screening, were also negative. He was subsequently transferred to the University of Alberta Hospital for further review and management. Additional recommended testing following his transfer of care included screening for antiphospholipid, lupus anticoagulant and hepatitis B core antibodies. All were negative. The transfusion history confirmed that the patient had previously received packed cell transfusions perioperatively more than 2 yrs prior to the present illness. The patient deteriorated with development of purpuric skin lesions and epistaxis while the progressive drop in the platelet count continued. The onset of purpuric bleeding followed red cell transfusion given during hemodialysis as a result of the acute renal shut down. At the time of the referral, he was on methylprednisolone and tranexamic acid. A review of his CBC showed hemoglobin; 88 g/L and total white cells; 14.2 × 10^9^/L (neutrophils 9.1). At the nadir, platelet count was 1 × 10^9^/L. The international normalized ratio (INR) fluctuated between 1.1 at admission and 2.1. PTT was stable and within normal limits. A summary of the CBC values and transfusion dates is given in Table 1. Review of peripheral blood film confirmed marked reduction in platelets with no evidence of microangiopathy or morphologic atypia. Histocompatibility tests reported patient as having HLA antibodies with panel reactive antibody (PRA) of 97%. The patient was homozygous for HPA-1a and HPA-4a and had alloantibodies against HPA-1b (anti-HPA-1b) demonstrated by ELISA with strong reactivity, OD ratio >7.0 (ELISA kit GTI, Inc., Waukesha, WI) (see Table 2). He received two doses of intravenous immunoglobulin (IVIG). Further transfusions of platelets were only from HLA matched donors who were HPA-1b homozygous only. At the time of discharge, his platelets had increased to 64 × 10^9^/L.  Figure 1 shows the trend of platelet count for the duration of admission. There was no further evidence of active bleeding or purpura and his hematologic profile gradually returned to normal values. He has remained clinically stable after discharge.

## 3. Discussion

Posttransfusion purpura is an immune mediated transfusion reaction with a low incidence in transfusion recipients. The clinical presentation results from severe thrombocytopenia and may include purpuric skin lesions, epistaxis, and mucosal bleeds. Bone marrow aspiration cytology (if performed) typically shows normal or increased megakaryocytes [1]. The classical presentation has the onset of thrombocytopenia within one week of the inciting transfusion and usually is unresponsive to platelet transfusion [4, 5]. It occurs as a result of antibodies directed against the platelet antigens, most commonly HPA-1a [4], but HPA-1b, HPA-3a and HPA-3b and HPA-4b antibodies have all been reported either singly or in combination as the culprit antibodies in case reports. This suggests that a wider serological spectrum should be evaluated in suspect cases. Furthermore antibody specificities against the platelet glycoprotein IIb/IIIa (previously called Zwa and Zwb) antigens have also been documented in posttransfusion purpura [6, 7]. The anti-HPA antibodies are of the IgG1 and IgG3 subclasses [7, 8]. It has been hypothesized that the initial blood transfusion triggers an anamnestic response boosting HPA antibodies in an already sensitized individual; however passive transfer of HPA antibodies has also been reported in rare situations [9]. The mechanism by which HPA alloantibodies destroy both autologous and platelets of donor origins contributing to the thrombocytopenia is not completely understood, but the development of nonspecific pan reactive antibodies against platelet glycoproteins may be responsible for the autologous platelet destruction in PTP [10]. In addition to its early recognition, prompt institution of definitive management is critical to patient survival.

Random platelet transfusion is generally unhelpful in the management of this condition as the platelet count drops further even with platelet transfusions. The response to platelet transfusion in our patient was poor, as expected. The patient was managed with intravenous immunoglobulin (IVIG) at 2 g/kg in two divided doses and subsequently received only HLA matched apheresis platelets from donors known to be HPA-1a/HPA-1a. He gradually recovered and the platelet count was restored. Plasma exchange and steroids are two other management modalities used in the past, but an increase in platelet count is delayed when compared with IVIG. Plasma exchange may be considered if patient is refractory to IVIG. The risk of a rebound thrombocytopenia makes continuous platelet count monitoring imperative until return to normal values.

The incidence of PTP in Africans is very low [11], and to our knowledge, the present case is the first of anti HPA 1b mediated PTP reported in a transfusion recipient in Canada. It is unclear if there is an ethnic dimension to this presentation as virtually all cases reported in North America are in Caucasian patients with anti-HPA 1a as the usual culprit. Several studies have now confirmed the critical place of single nucleotide polymorphisms (SNPs) in determining clinically recognizable variations in racial phenotypic paradigms of some clinical syndromes, for example, benign ethnic neutropenia. An earlier study of the genetics of platelet antigens had localized the genetic determinant of HPA-1a and -1b to HPA-la single nucleotide polymorphism (SNP) on chromosome 17 (q21.32) [3]. The mutation results in the substitution of amino acid Proline for Leucine at position 33 of the GPIIIa. However the recognition of single base substitution as plausible explanation for unusual presentation of clinical syndromes at variance from an established paradigm might suggest race as a factor. The frequency of HPA-1a versus HPA-1b and/or other HPA phenotypes according to ethnicity might allow for prediction of etiologic antibodies in cases of PTP. Halle et al. reported the absence of HPA-1b antigens in some sub-Saharan population [12]. This submission will require further studies of identified cases for validation. Evaluation of HPA polymorphisms among different ethnic groups may also be informative.

## 4. Conclusion

Accurate diagnosis of posttransfusion purpura remains challenging and a high index of suspicion is key. Given that the correct identification of the offending antibody is pivotal to platelet transfusion support, anti-HPA-1b may be an important exclusion in a posttransfusion purpura diagnosed patient of African descent.

## Figures and Tables

**Table 1:** 

	Parameters				
	Hb (g/dL)	Platelet count (×10^9^/L)	WBC (×10^9^/L)	Fib	INR
At admission (05/03/2010)	14.8	138	7.3	2.3	1.1
Day 5 (05/08/2010)	8.4	34	6.3	7.9	2.1
Day 20 (05/23/2010)	7.8	22	8.5	3.5	1.4
Day 28 (05/31/2010)	7.9	11	9.6	4.4	1.2
Day 35 (06/08/2010)	8.8	1	14.2^#^	3.8	1.2
Day 45 (06/18/2010)	9.4	64	11.6	4.6	1.1

*Fib: fibrinogen.

^#^Numerous nucleated cells; corrected WBC 10.2.

**Table:** (a)

HPA-1	HPA-4	HPA-5	HPA-15
1a/1a	4a/4a	5a/5b	15a/15a

**Table:** (b)

HPA-1	HPA-3	HPA-4	GPIIb/IIa	HPA-5	GPIb/IX	HLA
1b	Negative	Negative	Negative	Negative	Negative	Positive
